# miR-215 promotes malignant progression of gastric cancer by targeting RUNX1

**DOI:** 10.18632/oncotarget.6736

**Published:** 2015-12-23

**Authors:** Na Li, Qi-Yue Zhang, Jian-Ling Zou, Zhong-Wu Li, Tian-Tian Tian, Bin Dong, Xi-Juan Liu, Sai Ge, Yan Zhu, Jing Gao, Lin Shen

**Affiliations:** ^1^ Department of Gastrointestinal Oncology, Key Laboratory of Carcinogenesis and Translational Research (Ministry of Education), Peking University Cancer Hospital and Institute, Beijing, China; ^2^ Department of Pathology, Key Laboratory of Carcinogenesis and Translational Research (Ministry of Education), Peking University Cancer Hospital and Institute, Beijing, China; ^3^ Central Laboratory, Key Laboratory of Carcinogenesis and Translational Research (Ministry of Education), Peking University Cancer Hospital and Institute, Beijing, China

**Keywords:** miR-215, gastric cancer, malignant progression, RUNX1

## Abstract

**Objective:**

miR-215 was reported to be downregulated and functioned as a tumor suppressor in several cancers. In contrast, miR-215 was preferentially upregulated in gastric cancer (GC) according to our data. Thus, we studied the potential biological function of miR-215 in GC.

**Methods:**

miR-215 expression was measured in 77 paired GC tissues and adjacent non-tumor tissues. Biological functions of miR-215 were analyzed using cell viability, colony formation, migration, invasion, cell cycle, apoptosis and luciferase assays as well as via tumorigenicity and metastasis analysis.

**Results:**

miR-215 was significantly upregulated in 7 GC cell lines and 77 GC tissues compared to adjacent non-tumor tissues (*P* < 0.05), and miR-215 expression was greater in advanced GC (stage III/IV; *P* < 0.05). Ectopic expression of miR-215 in GES-1 and HGC-27 cells (low miR-215 expression) promoted cell growth, migration, invasion, and metastasis, and these were reversed in NCI-N87 cells (high miR-215 expression) after miR-215 downregulation. Potential target genes of miR-215 were predicted and RUNX1, a transcription factor and a tumor suppressor, was confirmed to be potential target according to luciferase studies. RUNX1 was downregulated in GC tissues compared to adjacent non-tumor tissues (*P* < 0.05), and RUNX1 reversed partial function of miR-215 *in vitro*.

**Conclusion:**

miR-215 promotes malignant progression of GC by targeting RUNX1, and RUNX1 can partially reverse miR-215 effects.

## INTRODUCTION

Gastric cancer (GC) is one of the three most common cancers in Japan, South Korea, and in China, where it is most prevalent and represents 47% of new annual cancer cases worldwide. Also, in China the mortality-to-incidence ratio is greater due to later stage diagnoses [1, 2]. Early detection and treatment as well as blocking or slowing GC malignancy progression are necessary to improve GC prognosis.

**Figure 1:**
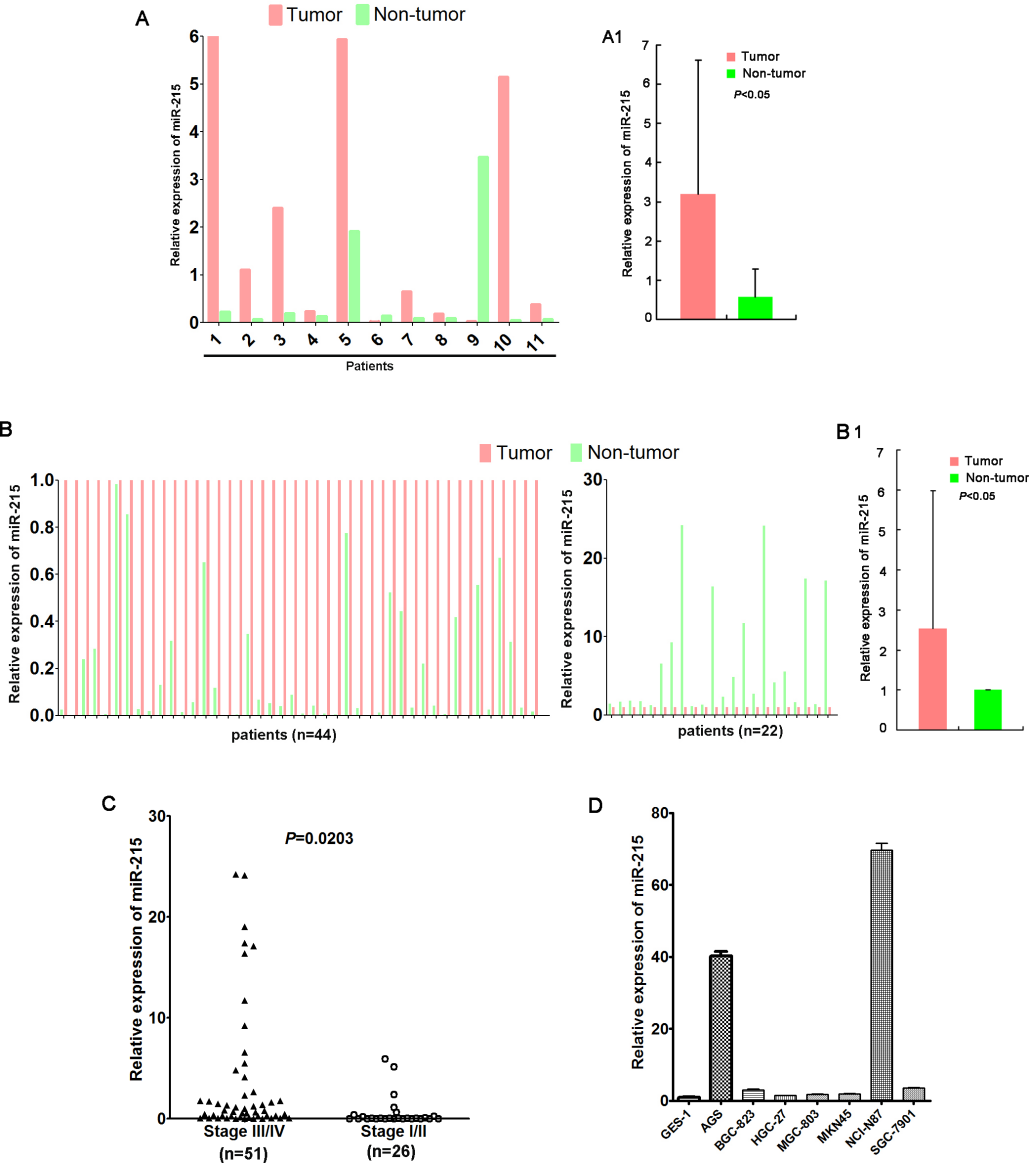
miR-215 expression was frequently up-regulated in GC miR-215 was frequently upregulated in GC tumor tissues compared with their matched non-tumor tissues according to quantitative PCR (**A, B**). (A) miR-215 was detected in a small discovery cohort (*n* = 11), and upregulated in 9 tumors compared to matched non-tumor tissues; (**A1**) The average expression of miR-215 of 11 tumors and matched non-tumor tissues presented in Figure 1A. (**B**) miR-215 expression was validated in a validation cohort (*n* = 66), and upregulated in 44 tumors compared to paired non-tumor tissues; (**B1**) The average expression of miR-215 of 66 tumors and matched non-tumor tissues presented in Figure 1B. miR-215 was normalized to internal control RNU6B. (**C**) Patients with stage III/IV GC had higher miR-215 expression than patients with stage I/II GC (*P* = 0.0203). (**D**) Compared to normal gastric epithelial cell line GES-1, miR-215 expression was up-regulated in seven GC cell lines.

Recently, researches have revealed that microRNAs (miRNAs) are important for the development and progression of multiple tumors including GC via regulation of several pivotal target genes [3–5]. In our previous study, we found that miR-215 was downregulated in colorectal cancer and patients with miR-215 high expression had lower 3-year relapse rate and longer median disease-free survival (DFS) compared with patients with miR-215 low expression [6]. Previous work suggested that miR-215 was downregulated and functioned as a potential tumor suppressor in several cancers including colorectal cancer [6–8] and esophageal adenocarcinoma [9]. But Deng Y et al. [10] performed miRNA microarray in six primary gastric tumors and their matched nonmalignant tissues and found that miR-215 was the most upregulated miRNA. As a result, we were interested in the role of miR-215 in GC. We found that miR-215 was preferentially upregulated in GC according to our data (Figure 1A), indicating a potential role for miR-215 in gastric carcinogenesis. Deng and colleagues reported that miR-215 was upregulated in GC and promoted cell proliferation *in vitro* via targeting RB1 and ALCAM [10, 11].

Understanding how miR-215 functions in GC may suggest potential molecular mechanisms of gastric carcinogenesis and progression as well as permit the development of novel therapeutic strategies for preventing or slowing GC. Thus, we measured miR-215 expression in paired GC tissues and adjacent non-tumor tissues and studied biological functions and a possible molecular basis of miR-215 in GC.

## RESULTS

### miR-215 was frequently up-regulated in gastric cancer

A total of 77 patients were enrolled (51 males; 26 females) with a median age of 60 years (range 32–80 years). Enrolled patients had stage III/IV GC (*N* = 51), liver metastasis (*N* = 20), and poorly differentiated tumors (*N* = 56).

Expression of miR-215 was measured in all 77 GC samples and adjacent non-tumor tissues using real-time PCR. Data show that miR-215 was frequently upregulated in 53 tumors (68.8%) compared to matched non-tumor tissues (*P* < 0.05; Figure 1A and 1B). miR-215 expression in patients with stage III/IV GC was significantly higher than in patients with stage I/II GC (*P* = 0.0203; Figure 1C). Finally, compared with normal gastric epithelial GES-1 cells, miR-215 expression was up-regulated in AGS, BGC-823, HGC-27, MGC-803, MKN45, NCI-N87, and SGC-7901 cells (Figure 1D).

### miR-215 promoted the growth of GC cells *in vitro* and *in vivo*

**Figure 2:**
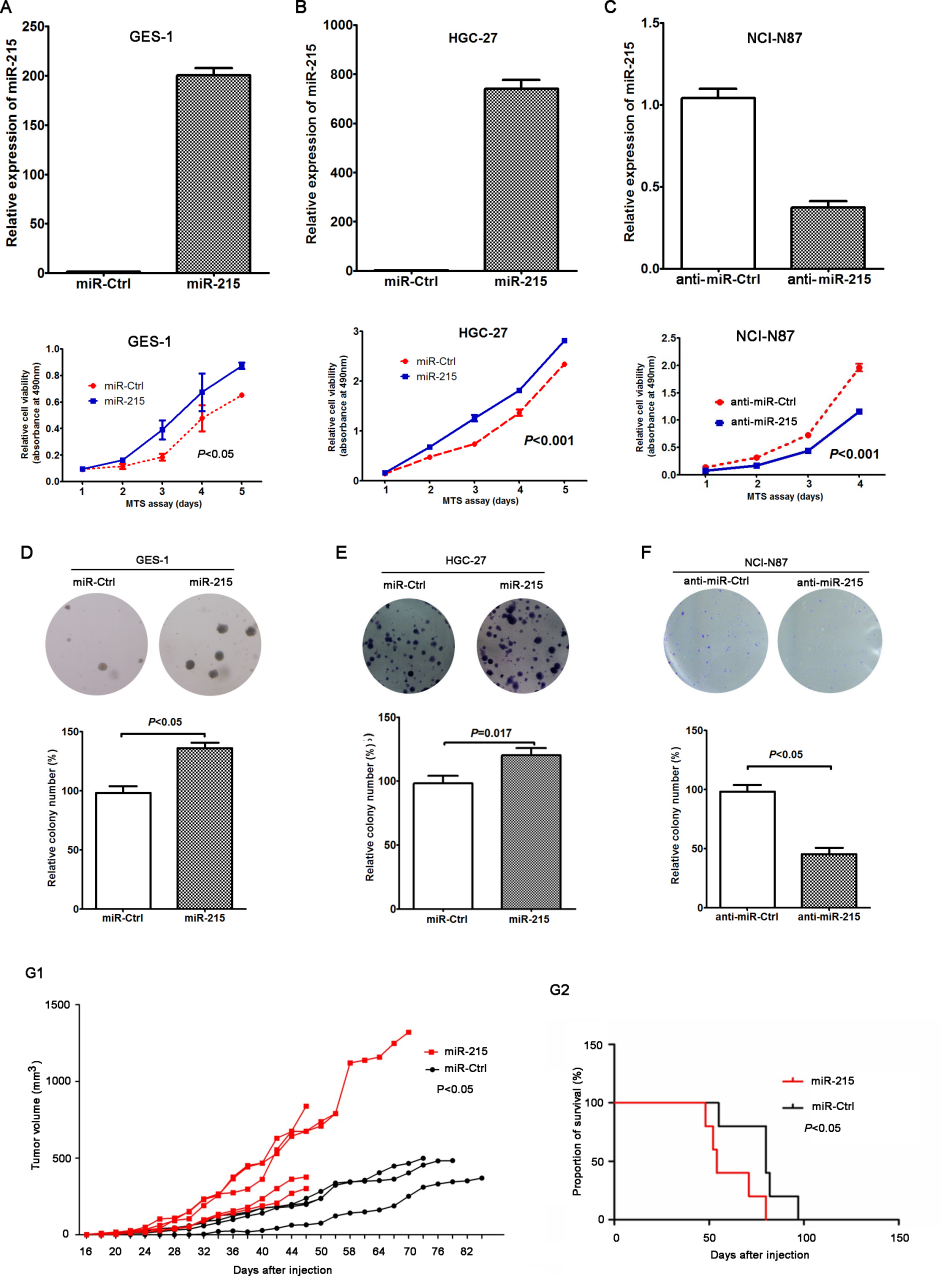
miR-215 promoted the growth of GC cells *in vitro* and *in vivo* Ectopic expression of miR-215 in GES-1 (**A**) and HGC-27 (**B**) cell lines promoted cell growth, but knockdown of miR-215 in an NCI-N87 cell line (**C**) inhibited cell growth. (**D**–**F**) Colony formation assay confirmed the function of miR-215 in GC cells. (**G1**) Tumor growth curve of xenografts derived from HGC-27 cells stably expressing miR-215 or control (*N* = 5 in miR-215 group; *N* = 4 in control group). (**G2**) OS curves of mice stably expressing miR-215 or control (*P* < 0.05).

Up-regulation of miR-215 in GC tissues suggested that miR-215 might act as an oncogene. To investigate the potential functions of miR-215 in GC cells, gain- and loss-of-function experiments were conducted by cell viability assays which showed that ectopic expression of miR-215 promoted cell growth in GES-1 (*P* < 0.05) and HGC-27 (*P* < 0.001) (Figure 2A and 2B); however, knockdown of miR-215 inhibited growth of NCI-N87 cells (*P* < 0.001; Figure 2C), and these data were confirmed by soft agar colony formation assay or usual colony formation assay (Figure 2D, 2E, and 2F).

HGC-27 cells with stable miR-215 expression or controls (miR-Ctrl) were injected subcutaneously into nude mice to establish *in vivo* xenografts. Data show that compared with the group of miR-Ctrl, tumor growth in HGC-27 cells with stable miR-215 expression was significantly accelerated (Figure 2G1; *P* < 0.05). Moreover, the overall survival (OS) was shorter for xenograft mice with stable miR-215 expression compared with mice with stable miR-Ctrl expression (median OS: 59 vs. 70 days; Figure 2G2; *P* < 0.05).

### miR-215 promoted migration, invasion and metastasis of GC cells *in vitro* and *in vivo*

**Figure 3:**
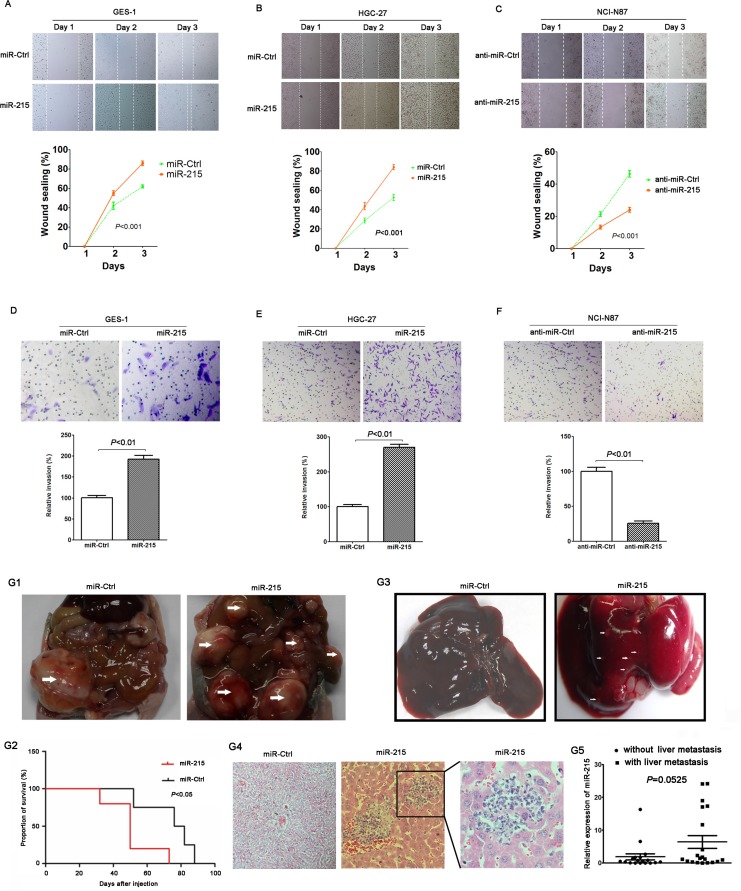
miR-215 promoted cell migration, invasion and metastasis *in vitro* and *in vivo* Ectopic expression of miR-215 in GES-1 and HGC-27 cell lines promoted cell migration (**A** and **B**) and invasion (**D** and **E**), meanwhile, knockdown of miR-215 in NCI-N87 cell line inhibited cell migration and invasion (**C** and **F**). (**G1**) Intraperitoneal tumors derived from HGC-27 cells stably expressing miR-215 spread in the peritoneal cavity compared to control. White arrow indicates gross observation of tumors. (**G2**) Mouse OS curves stably expressing miR-215 or control (*P* < 0.05). (**G3**) Gross observation of the liver tissues from nude mice, white arrow indicates metastatic foci. (**G4**) Representative H & E stained liver sections containing metastatic foci. (**G5**) miR-215 in 20 patients with liver metastasis were higher than in patients without liver metastases (*P* = 0.0525).

Wound healing assay and transwell invasion assay were used to analyze the potential role of miR-215 in GC cell migration and invasion. Data show that compared to control cells, ectopic expression of miR-215 promoted migration and invasion of GES-1 (Figure 3A and 3D; *P* < 0.01) and HGC-27 cells (Figure 3B and 3E; *P* < 0.01). Moreover, knockdown of miR-215 in NCI-N87 cells inhibited cell migration and invasion compared with control cells (Figure 3C and 3F; *P* < 0.01).

HGC-27 cells stably expressing miR-215 or control were injected into the peritoneal cavity of nude mice. Data show that compared with the control group, intraperitoneal tumors with stable miR-215 expression spread in the peritoneal cavity (Figure 3G1), and mice with stable miR-215 expression had poorer OS (median OS: 54 vs. 80 days; Figure 3G2; *P* < 0.05). Livers excised from mice and compared to controls (no metastatic focus was found in 5 mice) revealed that metastatic foci in livers with stable miR-215 expression were increased (several metastatic foci were found in 3 mice; Figure 3G3 and 3G4). Of the 77 GC patients, 20 had liver metastasis and miR-215 was greater than for patients lacking liver metastases (Figure 3G5; *P* = 0.0525). These intriguing data should be validated in studies of larger samples.

### miR-215 inhibited the expression of RUNX1 via binding to its 3′UTR

**Figure 4:**
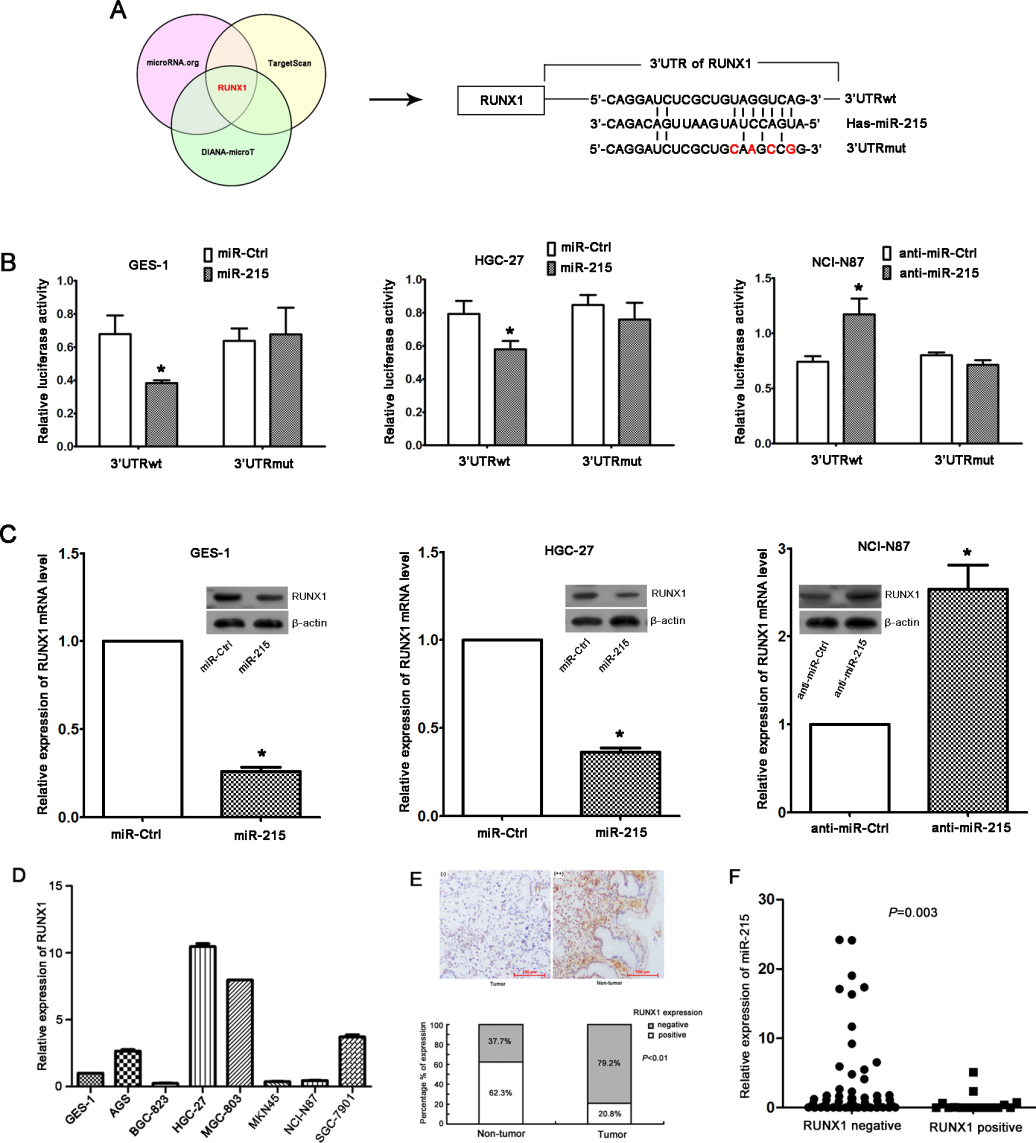
RUNX1 is a direct target of miR-215 (**A**) RUNX1 was predicted as a target gene of miR-215 by three prediction software packages, and the potential binding site of miR-215 in 3′UTR of RUNX1 was presented. 3′UTRwt, wild-type region of 3′UTR; 3′UTRmut, mutant region of 3′UTR. (**B**) Luciferase reporter assays in GES-1, HGC-27, and NCI-N87 cell lines indicated that miR-215 could only bind the wild-type region of 3′UTR of RUNX1, which suggested RUNX1 was a direct target gene of miR-215. Each experiment was repeated at least three times in triplicate, and data are means ± SD. *P < 0.05. (**C**) Ectopic expression of miR-215 in GES-1 and HGC-27 cell lines reduced RUNX1 mRNA level and protein. Likewise, knockdown of miR-215 in NCI-N87 cell line increased RUNX1 mRNA and protein. (**D**) RUNX1 expression in seven GC cell lines and one normal gastric epithelial cell line GES-1. (**E**) Immunohistochemistry of RUNX1 expression in GC tissues and non-tumor tissues (magnification, ×100), and percentage of positive or negative staining for RUNX1 protein in 77 paired GC tissues and adjacent non-tumor tissues are shown. (**F**) RUNX1 expression in GC tissues was inversely correlated with miR-215 expression (*P* = 0.003).

Three prediction software packages, miRNA, TargetScan, and DIANA-microT were used to identify miR-215 target genes and potential binding sites in the 3′UTR of RUNX1 (Figure 4A). To validate the specific regulation of miR-215 on RUNX1, luciferase reporter assays were performed followed by successful construction of pMIR-REPORT vectors containing wild-type or mutant seed regions of RUNX1. Data show that ectopic expression of miR-215 decreased luciferase activity of wild-type RUNX1 3′UTR and had no effect on mutant RUNX1 3′UTR in GES-1 and HGC-27 cell lines or in the NCI-N87 cell line (Figure 4B). Thus, RUNX1 is a direct target of miR-215 through binding to 3′UTR of RUNX1.

We confirmed that ectopic expression or knockdown of miR-215 can reduce or increase of RUNX1 mRNA and protein expression, respectively (Figure 4C). Moreover, RUNX1 expression in 7 GC cell lines (Figure 4D) and 77 paired GC and non-tumor tissues (Figure 4E) was measured with real-time RT-PCR and immunohistochemistry, respectively. We observed that 20.8% of GC tissues stained positive for RUNX1 protein which was significantly lower than that observed in adjacent non-tumor tissues (62.3% *P* < 0.01; Figure 4E), suggesting that RUNX1 expression was down-regulated in GC tissues, and was inversely correlated with miR-215 expression (Figure 4F; *P* = 0.003). However, no significant correlations were found between RUNX1 expression and disease stage or liver metastasis (data not shown), which might be due to the limited samples and would be validated in future with larger samples.

### RUNX1 could reverse partial function of miR-215 *in vitro*

**Figure 5:**
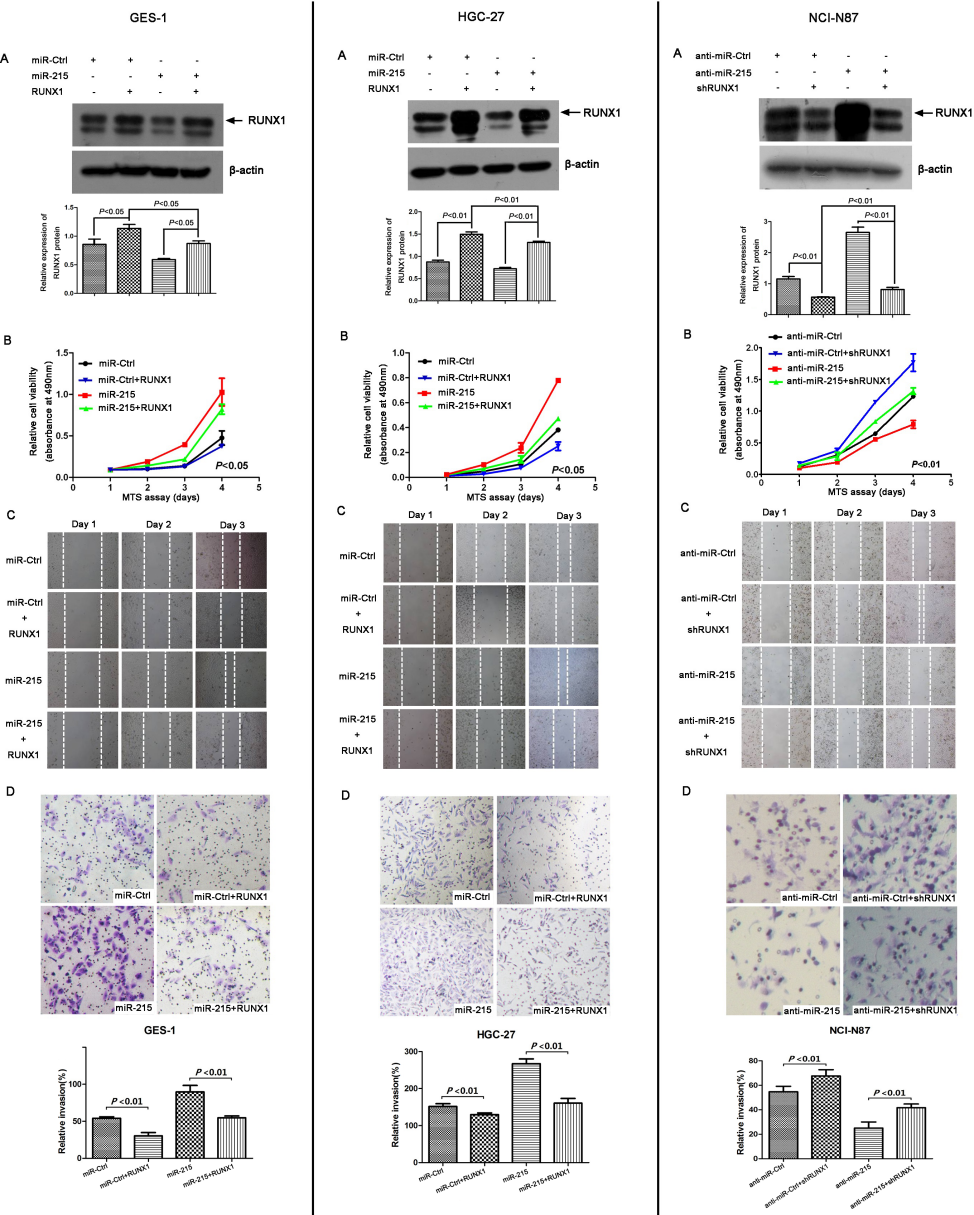
RUNX1 can reverse partial functions of miR-215 *in vitro* (**A**) RUNX1 was measured by Western blot in GES-1 and HGC-27 cell lines after co-transfection with miR-215/miR-Ctrl with or without RUNX1 for 48 h, and in NCI-87 cell line after co-transfection with anti-miR-215/anti-miR-Ctrl with or without shRUNX1 for 48 h. Cell viability (**B**), cell migration (**C**), and cell invasion (**D**) were analyzed by MTT, wound healing, and Matrigel invasion assays, respectively. Data show that ectopic expression of RUNX1 decreased cell proliferation, migration, and invasion induced by miR-215 in GES-1 and HGC-27 cell lines, and knockdown of RUNX1 increased cell proliferation, migration, and invasion compared to knockdown of miR-215 alone in NCI-N87 cell line.

miR-215 may promote malignant progression of GC and RUNX1 was a direct target of miR-215, so we analyzed the interactions of miR-215 and RUNX1 with a rescue assay *in vitro*. RUNX1 was measured with Western blot after ectopic expression of miR-215 and RUNX1 in GES-1 and HGC-7 cell lines or knockdown of miR-215 and RUNX1 in NCI-N87 cell line (Figure 5A). Figures 2 and 3 show that miR-215 can promote cell proliferation, migration, and invasion. Moreover, compared with ectopic expression of miR-215 alone, cell proliferation, migration, and invasion was reduced with co-transfection of miR-215 and RUNX1 in GES-1 and HCG-27 cell lines (Figure 5B–5D). Likewise, compared to knockdown of miR-215 alone, cell proliferation, migration, and invasion increased with co-transfection of anti-miR-215 and shRUNX1 in an NCI-N87 cell line (Figure 5B–5D). Thus, RUNX1 may reverse partial function of miR-215 *in vitro*.

## DISCUSSION

MicroRNAs (miRNAs) are important epigenetic regulators in the development and progression of human malignancies [5, 12–14]. Abnormal expression of various miRNAs has been reported in GCs, acting as oncogenes or tumor suppressors [15, 16]. miR-215 is downregulated and acts as a tumor suppressor in several cancers such as colorectal cancer [6, 17], breast cancer [18], and esophageal adenocarcinoma [9]. Reports of miR-215 expression in GC have been inconsistent [7, 17–19]; however, miR-215 was shown to be preferentially upregulated in GC in our work (Figure 1), indicating a potential role of miR-215 in gastric carcinogenesis, which was consistent with the results published by others [10, 11]. Also, miR-215 expression was higher in advanced GCs (stage III/IV; Figure 1; *P* < 0.05), indicating a potential role of miR-215 in the progression of GC.

To validate the potential role of miR-215 in the development and progression of GC, *in vitro* cell and *in vivo* animal experiments were conducted. Data show that miR-215 can promote cell proliferation, colony formation, cell migration and invasion *in vitro*, and can induce tumorigenesis and liver metastasis *in vivo* (Figures 2 and 3). Our work offers more details about miR-215 than previous reports. Deng and colleagues reported that miR-215 can promote cell proliferation *in vitro* [10] and Jin's group reported that miR-215 facilitated cell proliferation and migration, not cell invasion, only in one GC cell line [11]. Our results indicate that miR-215 may be a diagnostic marker and a therapeutic target.

Based on *vivo* tumorigenicity and metastasis assays, mice stable expressing miR-215 has a significantly shorter OS than the controls, indicating that miR-215 might be a prognostic factor of GC. Because of the limited samples and confounding factors, the correlation between miR-215 expression and patient prognosis was not analyzed, but it will be included in future studies.

To identify possible mechanisms involved in miR-215′s ability to promote cell cycle and apoptosis. FACS analysis was used to confirm ectopic expression of miR-215 in GES-1 and HGC-27 cell lines had no effect on the cell cycle (Supplementary Figure 1A and 1B); however, knockdown of miR-215 in NCI-N87 cell line revealed a slight increase of cells in the S phase compared with controls (Supplementary Figure 1C), which inhibited mitosis. Concomitant with cell cycle arrest at the S phase, upregulation of protein expression of p53 and p21, and downregulation of cyclin D1 and CDK2 occurred (Supplementary Figure 1C). It was well known that p53 induced cycle arrest by upregulating CDK inhibitor p21 and p27, which played important roles in cell cycle arrest. Meanwhile, cyclin D1-CDK2 complex attenuation was a pivotal factor for S cell cycle progression. Thus, knockdown of miR-215 revealed a slight increase of cells in the S phase partially due to a mechanism involving upregulation of protein expression of p53 and p21, and downregulation of cyclin D1 and CDK2. Neither ectopic expression nor knockdown of miR-215 significantly affected cell apoptosis in this study (Supplementary Figure 1D), which was consistent with the results presented by Deng [10] and Jin's group [11]. Thus, miR-215 does not participate in GC via apoptosis, but its effect on the cell cycle is discordant in different studies, which was needed to be further confirmed.

Several target genes regulated by miR-215 in different cancers have been reported [10, 20–22], and we observed that RUNX1 is a direct target of miR-215 as evidenced by the fact that ectopic expression of miR-215 reduced luciferase activity of the RUNX1 promoter and miR-215 downregulated RUNX1 expression (Figure 4). RUNX1 was reported to be a transcription factor and a tumor suppressor [23, 24], which we validated in GC, and we observed that partial functions of miR-215 could be rescued. Also RUNX1 was regulated by other miRNAs, such as miR-27a and miR-675 [25, 26] and several important target genes were confirmed to be regulated by miR-215. The tumor suppressor, RB1, was confirmed to be regulated by miR-215 in GC [10], and we validated that ectopic expression of miR-215 in GES-1 and HGC-27 cell lines decreased expression of RB1 (Supplementary Figure 2A and Supplementary Figure 2B), and knockdown of miR-215 in an NCI-N87 cell line increased RB1 expression (Supplementary Figure 2C).

As a transcription factor, RUNX1 could regulate other pathways directly or indirectly, such as transforming growth factor-β (TGF-β) signaling, bone morphogenetic protein (BMP) signaling, and wnt signaling pathways [27, 28]. In the future study, investigations would be conducted to explore the relationship between RUNX1 and its downstream genes. It was well known that RUNX1 was first identified in acute myeloid leukemia and played important roles in regulating the functions of hematopoietic cells [29, 30]. Extensive studies of RUNX1 had been conducted in leukemia and haematopoiesis, and RUNX1 was recognized as a suppressor for leukemogenesis. Along with more and more studies in different fields, RUNX1 was identified to play different roles in other tumors. RUNX1 functioned as a tumor suppressor in oesophagus cancer and gastric cancer, however, in oral squamous cell carcinomas and head and neck squamous cell carcinomas, RUNX1 played tumorigenic functions [31]. Further studies were needed to be performed to investigate the role of RUNX1 in different tumors.

In conclusion, we investigated biological functions of miR-215 in GC for the first time, and found that miR-215 was upregulated in GC and could promote malignant progression of GC cancer by targeting RUNX1, suggesting that miR-215 may function as a diagnostic marker and a therapeutic target.

## MATERIALS AND METHODS

### Gastric samples and cell lines

Seventy-seven pairs of GC tissues and adjacent non-tumor tissues were obtained from Peking University Cancer Hospital from August 2010 to October 2013. All patients were confirmed to have gastric adenocarcinoma and formalin-fixed paraffin-embedded surgical tumor or adjacent non-tumor tissue biopsy tissue samples collected prior to therapy. Clinicopathological characteristics of patients were collected from medical records and all patients gave written informed consent for their tissues to be used in research. This study was approved by the Ethics Committee of Peking University Cancer Hospital.

Seven GC cell lines, AGS, BGC-823, HGC-27, MGC-803, MKN45, NCI-N87, SGC-7901, and one normal gastric epithelial cell line (GES-1) were used. All cell lines were cultured in RPMI 1640 medium (Gibco BRL, Carlsbad, CA, USA) supplemented with 10% fetal bovine serum (Gibco BRL) and incubated in a humidified 37°C incubator supplemented with 5% CO_2_.

### RNA extraction and quantitative real-time PCR

Total RNA was extracted from tissues and cells using miRNeasy FFPE Kit (Cat. No.217504, Qiagen) and Trizol reagent (Invitrogen, Carlsbad, CA, USA) according to the manufacturer's instructions. RNA samples OD_260_/OD_280_ ratios ranging from 1.9–2.0 were considered good quality. Reverse transcription and quantitative PCR measurements for miR-215 and endogenous control RNU6B were performed with TaqMan MicroRNA Assays (Applied Biosystems, Foster City, CA). Relative expression of miR-215 was calculated using a comparative CT method.

Reverse transcription and quantitative PCR for RUNX1 mRNA were conducted using random primer using ABI^®^ Reverse Transcription Kit (Applied Biosystems, Foster City, CA) and SYBR Green master mixture (Applied Biosystems) with the housekeeping gene GAPDH as an internal control. RUNX1 primers were as follows: forward 5′-AATGCTACCGCA GCCATGAAG-3′, reverse 5′-GGTTTGTGAAGACAGTGA TGGTCAG-3′.

### Cell viability assay

Cells were transfected with miR-215/miR-Ctrl, anti-miR-215/anti-miR-Ctrl (GenePharma, Shanghai, China), pIRES2-EGFR-RUNX1 (kindly provided by Professor Yixue Xue from China Medical University), and pSuper-RUNX1-shRNA (kindly provided by Professor Fengming Luo from West China Hospital of Sichuan University) using Lipofectamine 2000 (Invitrogen). After 48 h of transfection, cell viability was measured using an MTT assay (Promega, Madison, WI) according to the manufacturer's instructions. Absorbance was measured at 490 nm using a spectrophotometer once a day for 4 consecutive days. MTT assay data were calculated relative to day 1.

### Colony formation assay

Cells (15 × 10^4^/well) were plated in a 6-well plate and transfected with miR-215, anti-miR-215 or control (miR-Ctrl/anti-miR-Ctrl). After 48 h of transfection, cells were subcultured in a new 6-well plate (500–1,000/well) for 14 days. Colonies were stained with 5% crystal violet and counted. All experiments were performed in triplicate wells.

### Cell migration assay

After cells were transfected with miR-215/miR-Ctrl, anti-miR-215/anti-miR-Ctrl, pIRES2-EGFR-RUNX1 and pSuper-RUNX1-shRNA for 48 h, cell migration was measured with a wound-healing assay in which the distance of two flanks of the wound was monitored once daily for 3 days.

### Cell invasion assay

Cells were transfected with miR-215/miR-Ctrl, anti-miR-215/anti-miR-Ctrl, pIRES2-EGFR-RUNX1, and pSuper-RUNX1-shRNA for 48 h, and cell invasion was measured using a Matrigel invasion assay (BD Biosciences, Erembodegem, Belgium). Cells in the upper compartment of the chamber were suspended in serum-free medium, and the lower chamber contained medium supplemented with 20% fetal bovine serum. After 24 h incubation, cells that passed through the matrigel membrane were fixed and stained with crystal violet and counted in 5 random microscopic fields.

### Soft agar colony formation assay

In brief, the base agar was prepared through mixing 1.5 ml 2X RPMI 1640 medium with 1.5 ml of 1.0% noble-agar (Sigma, St. Louis, MO, USA) solution. After solidification of base agar, the top agarose prepared by mixing 0.5 ml 2X cell suspension (0.5 × 10^4^ cells) with 0.5 ml of 0.7% agarose solution was added to the base agar followed by incubation at 37°C for 14 days. All experiments were performed in triplicate wells and colonies were counted under a microscope.

### Cell cycle assay

After transfection with miR-215/miR-Ctrl and anti-miR-215/anti-miR-Ctrl for 48 h, cells were harvested and fixed in 70% cold ethanol for at least 12 h at 4°C. Cells were stained with 50 μg/mL propidium iodide (BD Biosciences) at room temperature for 30 min in the dark, and the cell cycle was assessed with a FACS Calibur system (BD Biosciences) and data were analyzed with ModFit 3.0 software (BD Biosciences).

### Annexin V apoptosis assay

Cell apoptosis was measured via Annexin V-Allophycocyanin (APC) and 7-amino-actinomycin (7-AAD) staining (BD Biosciences) for 15 min at room temperature in the dark, followed by flow cytometry within 1 h (BD Biosciences). Cell apoptosis was measured using WinMDI 2.9 software (BD Biosciences).

### Western blot

Total protein was extracted from cell pellets using CytoBuster Protein Extraction Reagent (Merck Millipore, Darmstadt, Germany). Protein was measured with a BCA Protein Assay Kit (Beyotime Biotechnology, Jiangsu, China), and 10 to 30 mg of protein from each sample was separated via 12% SDS-PAGE. After protein transfer to nitrocellulose membranes (GE Healthcare, Piscataway, NJ) samples were incubated with primary antibody at 4°C overnight and secondary antibody at room temperature for 1 h (antibodies are depicted in Supplementary Table S1). Proteins were visualized with ECL Plus Western Blot Detection Reagents (GE Healthcare).

### *In vivo* tumorigenicity

Stable miR-215-expressing and anti-miR-215-expressing cell lines and control cell lines were generated with lentiviral infection (GenePharma, Shanghai, China) according to the manufacturer's protocol. HGC-27 cells (2 × 10^6^) stably expressing miR-215 or control and NCI-N87 cells (2 × 10^6^) stably expressing anti-miR-215 or control were injected subcutaneously into the dorsal right flank of 6-week-old female Balb/c nude mice (*N* = 5 mice/group). Tumors and animal weights were measured twice every week from the first injection until animal sacrifice and tumor volumes were calculated using the following formula: V = (L × W^2^)/2 (V, volume; L, length; W, width of tumor). All animal experiments were performed according to the animal experimental guidelines of Peking University Cancer Hospital.

### *In vivo* metastasis assay

HGC-27 cells (2 × 10^6^) stabling expressing miR-215 or control and NCI-N87 cells (2 × 10^6^) stably expressing anti-miR-215 or control were injected into the peritoneal cavity of 6-week-old female Balb/c nude mice (*N* = 5 mice/group). Mouse weight was measured twice every week from the first injection animal sacrifice. Mouse livers were excised and embedded in paraffin for hematoxylin and eosin staining.

### Luciferase reporter assay

The potential miR-215-binding sites in RUNX1 3′ untranslated region (3′UTR) were predicted by TargetScan, miRanda (www.microRNA.org), and DIANA-microT. Sequences containing wild-type or mutant seed region of RUNX1 (Figure 4B) were synthesized and cloned into pMIR-REPORT luciferase vector (Applied Biosystems). Cells in 24-well plates were co-transfected with miR-215/anti-miR-215 or control, pMIR-REPORT vector, and pRL-TK vector using Lipofectamine 2000 (Invitrogen). After transfection for 48 h, luciferase activity was measured using a dual-luciferase reporter assay system according to the manufacturer's instructions (Promega).

### Immunohistochemistry for RUNX1

FFPE sections (4 μm) of GC tissues and adjacent non-tumor tissues were deparaffinized in xylene and hydrated in a graded alcohol, followed by retrieval in 10 mmol/L citrate buffer (pH 6.0) and endogenous peroxidase treatment with 3% H_2_O_2_. After incubation with 5% BSA for 45 min, sections were then incubated with RUNX1 antibody (dilution: 1:350; Abcam, city, state) at 4°C overnight. After incubation with secondary antibody, signal production was conducted using Dako EnVision System (Dako, Glostrup, Denmark). Sections were scored by two independent professional pathologists from the pathology department of present hospital who were blinded to this study. Staining was graded as −, +, ++, and +++, and expression was considered positive or negative based on the median staining score.

### Statistical analysis

Statistical analysis was performed using GraphPad Prism 5 software (GraphPad Software, Inc., city, state). A Student's *t*-test was used to assess differences between groups. Differences in cell growth curves and *in vivo* tumorigenicity were confirmed with repeated-measures ANOVA. The difference in miR-215 expression between tumor and non-tumor tissues was compared using a Mann-Whitney *U*-test. Data are means ± SD. *P* < 0.05 was considered statistically significant.

## SUPPLEMENTARY MATERIALS FIGURES AND TABLE


