# Supplementary material for: miR-215 promotes malignant progression of gastric cancer by targeting RUNX1
Source: Oncotarget. 2015 Dec 23;7(4):4817–28. doi: 10.18632/oncotarget.6736 (PMC4826245; doi:10.18632/oncotarget.6736)
Supplement: Supplementary file 1 [file oncotarget-07-4817-s001.pdf]

miR-215 promotes malignant progression of gastric cancer by targeting RUNX1

Supplementary Materials

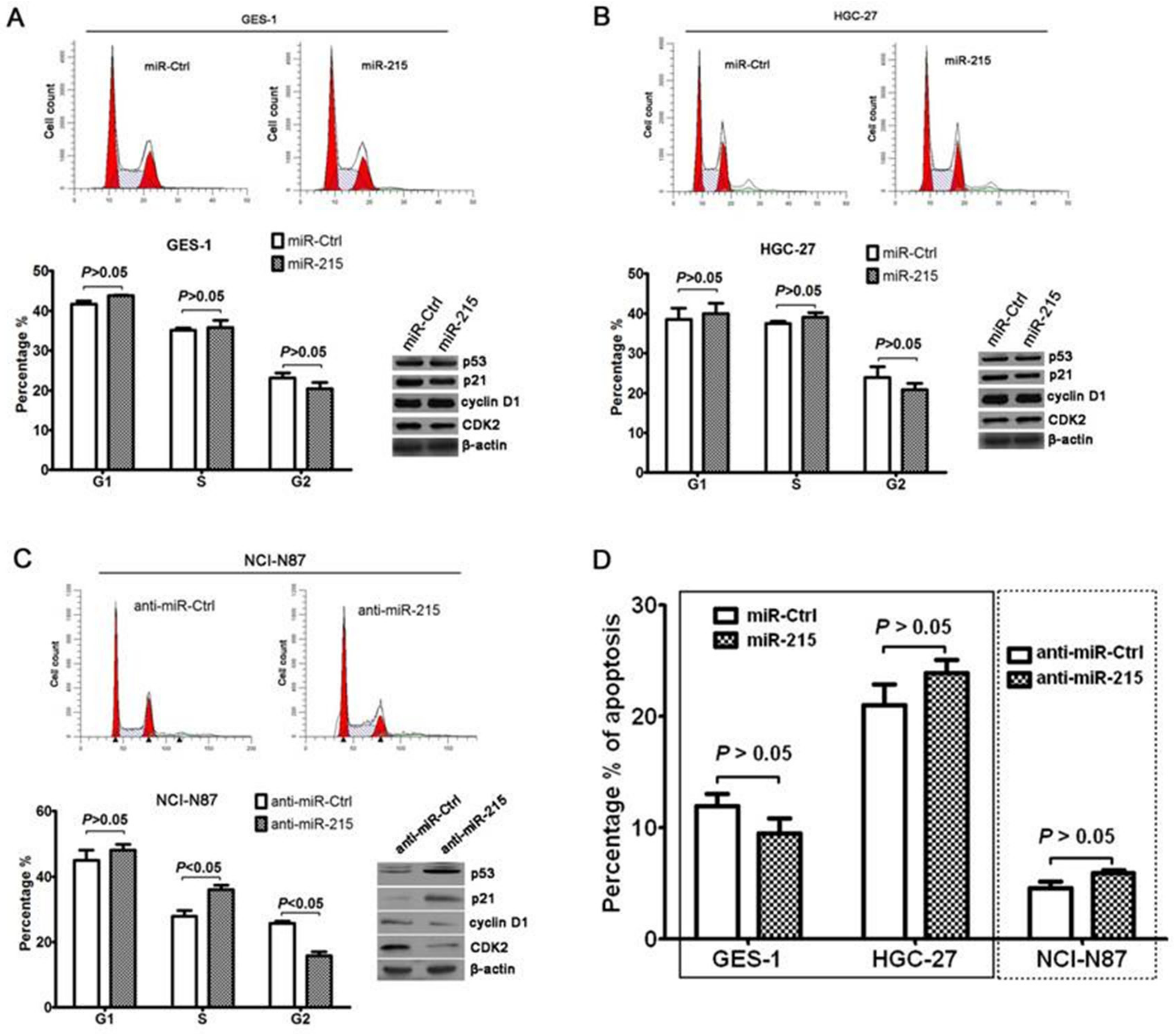

Supplementary Figure S1: The effects of miR-215 on cell cycle and cell apoptosis. Ectopic expression of miR-215 in GES-1 (**A**) and HGC-27 (**B**) cell lines had no effect on cell cycle, however, knockdown of miR-215 in NCI-N87 cell line revealed a slight increase of cells in S phase compared to control. (**C**) Concomitant with cell cycle arrest at S phase, the upregulation of protein expression of p53 and p21, and downregulation of cyclin D1 and CDK2 were found (C). (**D**) Neither ectopic expression nor knockdown of miR-215 had significant effect on cell apoptosis ($P > 0.05$).

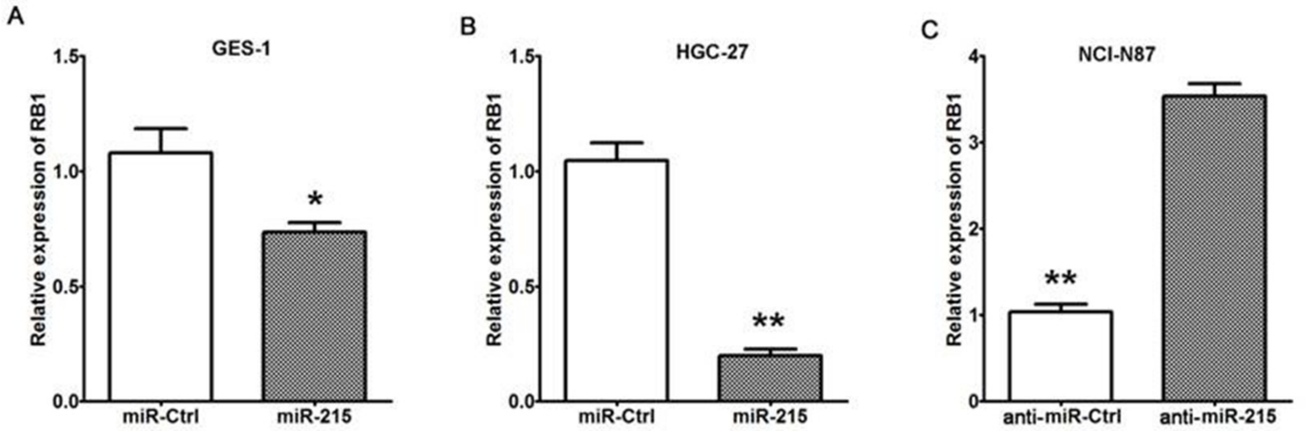

Supplementary Figure S2: RB1 is regulated by miR-215. Extopic expression of miR-215 in GES-1 (**A**) and HGC-27 (**B**) cell lines decreased the expression of RB1, and knockdown of miR-215 in NCI-N87 cell line increased RB1 expression (**C**). *$P < 0.05$, **$P < 0.01$.

Supplementary Table S1: Antibodies used in this study

Antibody	Company	Catalog	Dilution	Source
P53	Cell Signaling	2527	1:750	Rabbit
P21	Cell Signaling	2947	1:750	Rabbit
Cyclin D1	Cell Signaling	2978	1:1000	Rabbit
CDK2	Origene	TA502893	1:2000	Mouse
RUNX1	Abcam	ab54869	1:1000	Mouse
β-actin	Sigma	122M4782	1:5000	Mouse
